# Situational judgment using ethical reasoning in Saudi undergraduate pharmacy students

**DOI:** 10.1186/s12910-022-00768-x

**Published:** 2022-04-12

**Authors:** Fahad Saleh Alkhuzaee, Majid Ali, Khang Wen Goh, Yaser Mohammed Al-Worafi, Long Chiau Ming

**Affiliations:** 1 King Abdullah Medical City, Makkah, Saudi Arabia; 2 School of Life and Medical Sciences, University of Hertfordshire (hosted by Global Academic Foundation), New Administrative Capital, Cairo, Egypt; 3 Faculty of Information Technology, INTI International University, Nilai, Negeri Sembilan Malaysia; 4 College of Pharmacy, University of Science and Technology, Sana’a, Yemen; 5 College of Pharmacy, University of Science and Technology of Fujairah, Fujairah, UAE; 6 PAPRSB Institute of Health Sciences, Universiti Brunei Darussalam, Gadong, Brunei Darussalam

**Keywords:** Situational judgment, Ethical dilemmas, Ethical reasoning, Moral development, Professional development

## Abstract

**Introduction:**

There is a paramount need for moral development for pharmacists and pharmacy students to practice the patient-centered profession. We aimed to explore the current situational judgment utilizing ethical reasoning among undergraduate pharmacy students.

**Methods:**

A set of ten ethical dilemmas, representing potential real-life situations that the students come across in the university and may face in the future as a pharmacist were developed by a team of students, academic staff, and stakeholders. These ethical dilemmas were validated, checked for accuracy, and piloted. An online questionnaire was created consisting of these ten scenarios as open-ended questions and administered to fourth year and fifth year PharmD students in a public university located at the city of Mecca, Saudi Arabia, asking them how they would react in that situation. Responses of the participants were analyzed using thematic analysis independently by four researchers and inter-rater agreement were achieved through consensus.

**Results:**

Out of 205, 186 students completed the questionnaire with a response rate of 90.7%. Analysis and resulted in the generation of 32 codes, which were then categorized into seven overarching themes: student engagement, social and professional responsibility, academic integrity, legal obligation, moral obligation, signposting, and moral engagement and patient safety.

**Conclusions:**

Undergraduate pharmacy students experience complex state of mind in connection with ethical reasoning. The participants’ situational judgment were driven by cultural norm, authority, and responsibility. Student engagement is also affected by the state of mind and feelings of mutual trust, perceived cultural influence and peer pressure. The students were prone to seek help from university administrators or teachers when faced with situations in which they were helpless.

**Supplementary Information:**

The online version contains supplementary material available at 10.1186/s12910-022-00768-x.

## Background

Ethical reasoning is a decision-making process that involves forming situational judgments on what may be morally right. It enforces the individuals to weigh the benefit of their actions and their possible consequences [1]. This definition is based on Lawrence Kohlberg’s cognitive moral development (CMD) theory that states moral development is essentially a conceptualized moral judgment based on six developmental stages [1]. The theory further asserts that all individuals develop their cognitive level in a series of unidirectional and logical sequences and stages that cannot be bypassed. A study in psychology has applied this theory to healthcare professions, including the pharmacy profession, and found that healthcare practitioners’ actions depend on impactful ethical reasoning and follow the same decision-making process as suggested by Kohlberg’s theory [2, 3].

Based on Kohlberg’s theory, moral development can be reasonably measured or assessed. In 1982, Kohlberg illustrated a way to assess moral judgement using a method that they called the moral judgment interview (MJI) [4]. MJI is a semi-structured interview in which participants are asked about several moral or ethical dilemmas. Their responses are then compared with a list of arguments staged at different levels of cognitive development, and a final score based on specific criteria is given. This method is unfeasible as it requires intensive efforts for large-scale implementation.

Another validated method to estimate the level of moral development is the Defining Issues Test (DIT). It consists of five to 12 dilemmas, answered by rating or ranking statements related to the dilemmas. This is followed by computerized scoring. A more developed version of DIT was introduced by Rest. This method incorporates a “P” score that is “the relative importance a subject gives to principled moral considerations while deciding in moral dilemmas” [5].

The pharmacy profession is regulated by codes of ethics, institutional interest, professional mannerisms, and legal frameworks in many countries. This creates an intense pressure to act in a certain way in favor of or against any of the involved parties’ values, whether patients, institutions, or society, creating ethical dilemmas and causing moral distress where any misguided moral judgment may compromise patient safety [6]. It is especially prevalent in community pharmacies, as it represents a turning point from a product-focused to a patient-centered profession. Pharmacists working in this sensitive setting must develop their moral judgment, necessitating an ethical covenant between the pharmacist and patient to work toward their well-being. Cooper et al. reported in their study that approximately 60% of the community pharmacists had encountered at least one ethical issue, but a third of them could not recall such situations [7]. From these results, the researchers deduced that the work environment influences the type and frequency of ethical dilemmas that individuals in this profession face.

Besides, another qualitative study mentioned that business and commercial interests may have an ethical influence on pharmacists’ decision making; however, in contrast, Wingfield found that company policies were helpful to pharmacists when faced with predicaments involving ethical dilemmas [8, 9]. Nevertheless, studies indicate that pharmacists do not always adhere to best practices when they face ethical dilemmas, which highlights the importance of improving their moral judgment and ethical reasoning to provide the best care for their patients [10, 11]. Furthermore, Latif’s work on ethical reasoning concluded that the community pharmacy environment affects the ethical reasoning when community pharmacists’ scores were compared with pharmacy students who had not been exposed to the same environment [6, 12].

After Kohlberg conceived his CMD theory, he tried to apply it to the educational environment [13]. Kohlberg and Blatt discovered that the students exposed to ethically complex dilemmas at an early stage improve their moral thinking and attempt to adopt the examples of moral judgments that have been provided to them in the study as their own.

Ethical reasoning is teachable, according to Latif’s research [12]. In one of his studies, he examined students’ moral thinking before and after delivering a communication skills course to them. Students were found to score significantly higher than their pre-course results. Developing ethical reasoning is not intended to increase their knowledge; rather, it is intended to guide students’ thinking, develop their moral and situational judgment, and act as a qualitative marker of their thinking process [14].

Healthcare professionals dealing with patients in the frontline on daily basis in any healthcare setting are likely to face ethical issues in which they are required to make decisions based on their judgment Therefore, there is a paramount need for moral development for pharmacists and pharmacy students to practice their patient-centered profession that has the potential to expose them to moral and ethical issues daily [15]. Pharmacy curricula focus mainly on educational outcomes and less on the development of ethical reasoning in pharmacy students. This leaves the students and recent graduates at risk of making decisions that may not be ethically appropriate in clinical practice. Early identification of the students who may struggle with situational judgment in ethical dilemmas can allow the pharmacy colleges to provide support to these students for developing their ethical reasoning. In this study, we aimed to explore the ethical reasoning of undergraduate pharmacy students of a public university located at the city of Mecca using a qualitative thematic analysis method.

## Methods

In this study, we developed ten ethical dilemmas that were presented to the students via an online questionnaire. The students' responses were then analyzed using thematic analysis to gain insight into their moral and situational judgment. The study was reviewed and approved by the Institutional Review Board (IRB) at Umm Al-Qura University, Saudi Arabia (approval number: HAPO-02-K-0122020-06-404).

### Development of ethical dilemmas

Several moral reasoning ability or situational judgment tests are available and are based on the scoring system [15–17]. However, they do not cover the holistic transition from student experiences in the university through to professional practice. Taking into consideration that the aim of the study was to explore the students’ judgment, therefore, we decided to develop scenarios which are culturally relevant to the local practice.

A team comprising ten members (undergraduate and postgraduate students, recent graduates, preceptors as stakeholders, and two academic staff members) was established. The team was tasked to create a range of ethical dilemmas which an individual may face across his pharmacy career starting from the university as a student through the professional job as a pharmacist. The team developed 20 ethical dilemmas that were then reduced to 10 after several discussion sessions by the team members. The objective was to develop simple scenarios that are comprehensible by the students and represent potential real-life situations that the students come across in the university and may face in the future as a pharmacist. The final ten scenarios were then checked for authenticity and validity by another two academic staff members and piloted with another three undergraduate students. Minor adjustments were made in the wordings used in the scenarios. These scenarios are presented in Additional file 1.

### Sampling and data collection

An online questionnaire consisting of these ten scenarios was created using Google Docs. In the questionnaire, each scenario was written as an open-ended question asking the participants to explain how they would react in that situation. The use of open-ended questions allowed the participants to express their responses and judgments unreservedly [6]. Since Arabic language is our students' first language, we allowed them to attempt the questions in either English or Arabic language, based on their choice, to freely express their judgments. The link to the questionnaire was emailed to all fourth and fifth year PharmD students during summer break. We included these students because they were in the middle of their summer placements either in a community pharmacy or hospital and could relate these scenarios to real life. To maintain anonymity, students were informed that their identities would not be disclosed in any way. Any confidential data, including participants’ identifiable information, was stored securely and removed before the data analysis was conducted. All relevant data were extracted for the sole purpose of this study and were not shared by any third party.

### Data analysis

The thematic analysis of all the given responses of participants was conducted by the four researchers who have high competency in both English and Arabic languages. The initial pilot phase of analysis included familiarization with the data and the generation of relevant codes by screening 20 responses independently. All four researchers then met to discuss each code and resolve any discrepancies to have an inter-rater agreement. The full analysis phase included screening and analysing all given response, including the 20 piloted tested responses. The researchers worked in pair and each pair screened through half of the collected responses. This was followed by categorizing these codes into overarching themes independently. All four researchers then met again to agree on common themes whereby any discrepancy was resolved through iterative discussions. Moreover, authors (F.S.A., M.A., Y.M.A.) who were not part of the four researchers also checked through all the codes and final themes for accuracy and confirmed the coding conducted by the two separate teams were aligned. The analysis was facilitated throughout via NVivo Pro 12® for Windows (version 12.1) program.

## Results

### Demographic characteristics

Out of 205 fourth year and fifth year PharmD students, 186 completed the questionnaire (91% response rate). Thematic analysis of the participants' responses resulted in the generation of 32 codes, which were then categorized into seven overarching themes. These codes and themes were not predefined. The seven final themes agreed upon by the researchers were: student engagement, social and professional responsibility, academic integrity, legal obligation, moral obligation, signposting, and moral engagement, and patient safety. These codes and the themes are listed in Tables 1 and 2.

**Table 1:** Codes generated from the scenarios

Scenarios	Codes
Scenario 1	Attending the lecture
	The morality of signing for others in their absence
	State of mind Mutual trust Perceived cultural influence
	Contacting the teacher
Scenario 2	The morality of giving away the answers
	Fairness Peer pressure
	Helping others
Scenario 3	Respecting the teacher
	Academic honesty
	Social ethics
Scenario 4	Following the code of ethics
	Work policy and procedures
	University's help
	Dangers of taking the medication without a prescription
Scenario 5	Plagiarism
	Fairness to other students
	Taking responsibility as a leader
Scenario 6	Patient confidentiality and privacy
	Physician role
Scenario 7	Motivating the patient
	Sense of duty
	Medication adherence
Scenario 8	Physician involvement
	Dangers of taking the medication without a prescription
	Accountability
	Guilt complex
Scenario 9	Religion beliefs
	Respecting the work policies
Scenario 10	Influence on patient’s health
	Work policies and procedures
	Involvement of department administrators
	Accountability

**Table 2:** List of codes categorized into themes

Codes	Themes	Description of themes
State of mind Mutual trust Perceived cultural influence Peer pressure	Student engagement	Student’s perceived cultural difference and mutual trust as well as state of mind determined their engagement in the given scenario and thus affected their decision
Medication adherence Work policies and procedures Social ethics Accountability Taking responsibility as a leader	Social and professional responsibility	Students envisaged improving medication adherence as well as the professional development skills related to their work as social and professional responsibilities
Plagiarism Academic honesty Fairness Morality of signing for others in their absence Morality of giving away the answers Fairness to other students	Academic integrity	Students identified several aspects related to academic integrity such as plagiarism, signing attendance for fellow students, and cheating in exams/assignments, and regarded them as academic dishonesty
Patient confidentiality and privacy Respecting the work policies Physician role Attending the lecture	Legal obligation	Students identified several aspects (as evident from the codes) in their undergraduate studies and professional practice as binding legal obligations
Sense of duty Respecting the teacher Following the code of ethics Religious beliefs Guilt complex	Moral obligation	Students identified several aspects (as evident from the codes) in their undergraduate studies and professional practice as moral obligations
University's help Physician involvement Contacting the teacher Involvement of department administrators	Signposting	Students highlighted the resources (as evident from the codes) for whistleblowing and signposting especially when faced with situations in which they were helpless
Motivating the patient Dangers of taking a medication without a prescription Influence on patient’s health Helping others	Moral engagement and patient safety	Students regarded promoting better patient healthcare and patient safety as their social roles and the means of their engagement in society

### Undergraduate scenarios

In the undergraduate scenarios, the participants’ responses generally reflected that student’s perceived cultural influence and peer pressure as well as their state of mind played a role in how they may behave or act in any given situation. The researchers came across high percentage of responses expressed feelings of fairness, citing that providing the answers in assignments or marking an absent student as “present” is unfair and unethical to them and other colleagues.

### Professional practice scenarios

The researchers uncovered that high frequency of the participants preferred following their work policies and procedures when faced with a moral or ethical dilemma. The participants also highlighted social responsibility in several of their responses. Most of them iterated that they must serve the public and follow the socio-ethical constructs. Some participants acknowledged the importance of stepping up and taking responsibility, especially in situations where they are assigned leadership roles. Participants were more cautious when faced with situations where patient confidentiality and privacy were at stake. They asserted that following the rules and protecting the patient's confidentiality, whether as students in the university, interns, residents, or as pharmacists, is of prime importance to them.

## Discussion

### Theme 1: student engagement

The National Survey of Student Engagement (NSSE) that was established in 1998 in the United States and Canada, categorized student engagement into two aspects; one requires efforts from the students ("the amount of time and effort students put into their studies") and the second is dependent on the institution's approach to engage the students ("how the institution deploys its resources and organizes the curriculum and other learning opportunities to get students to participate in activities") [16]. According to this, the institution's increased and directed efforts can lead to better student engagement, which can affect their situational judgment in ethical dilemmas related directly or indirectly to their experiences in the university and the profession in the future.

Student engagement has also been described as the emotional investment in learning that is exerted by the student. At a fundamental level, engagement means allowing the students to participate actively and giving them the agency to do so [17]. The importance of active participation by learning with their peers and interacting with their teachers was highlighted by the participating students in our study. This is also iterated by the four factors illustrated by NSSE to identify how well-engaged the students are [18]. These factors include academic challenges, the amount of learning with peers, experiences with faculty members, and interactions with the campus environment. In agreement with the aforementioned factors, mutual trust, perceived cultural influence and peer pressure have been linked to student engagement and feelings, which can lead to the decisions they make [19–21].

### Theme 2: social and professional responsibility

In our study's professional practice scenarios, the participants stated that improving medication adherence is a social and professional responsibility of pharmacists. Moreover, the participants preferred following the work policies and procedures, highlighting the importance of healthcare organizations making newly employed staff aware of work policies and procedures. The participants also highlighted the social responsibility of adopting a leadership role when such a situation arises. Leadership skills have traditionally been expected of undergraduate pharmacy students. This is evident in the Accreditation Council for Pharmacy Education (ACPE) guidelines and standards. Most healthcare organizations recognize fostering this skill as a learning goal and professional development [22]. Studies have also shown that students who demonstrate a higher level of ethical reasoning with accountability are inclined to behave more professionally [23]. The pharmacy colleges and the stakeholders must consider the aspect of inculcating the elements of accountability and dependability in pharmacy students. The social responsibility of serving the public emerged in the majority of the 5^th^ year students' responses in our study. Moreover, the development of this characteristic has been reinforced by numerous educational organizations [24].

### Theme 3: academic integrity

While the act of students providing assignment or exam answers to their colleagues may be carried out in good faith, it insinuates academic dishonesty on the student’s part. The association between academic integrity and ethical reasoning among pharmacy students has been established by several studies [25], and this connection may have been generated from the notion that most students engage in this type of behavior due to a lack of self-control associated with potential opportunities obtained from such acts [26]. It is a common practice for students to provide answers to their peers. As demonstrated by an Australian study, 90% of undergraduate students consider it acceptable to have their assignments written by someone else [27]. However, participants in our study envisaged this act as well as signing in class on behalf of the absent colleagues unfair to themselves and other colleagues. These aspects were mainly seen in the responses of 5^th^ year students and could have been due to their maturity and commitment to their career compared to the other participants [28, 29]. Conversely, other studies found that senior students were less likely to identify instances of academic misconduct as compared to the other students [30–32].

The International Center for Academic Integrity (ICAI) describes academic integrity as a concept that encompasses five fundamental values (honesty, trust, fairness, respect, and responsibility) that are integral to most morally guided professions, such as medicine, pharmacy, nursing, and law [33, 34]. In 2013, the ICAI updated its original document to include one more value, courage [34]. In all undergraduate scenarios in our study, participants' responses were centered around fairness, respect, and responsibility, indicating the fact that academic integrity plays a part in their moral decision-making process. Several studies have examined and reported healthcare students' academic integrity with conflicting results [30, 31].

### Theme 4: legal obligation

A notable theme identified in participants’ responses in our study was that of the legal obligation. Participants’ responses to undergraduate scenarios reflected the importance of attending the lectures in the university without valid excuses. The responses to postgraduate scenarios highlighted the significance of respecting patient confidentiality and privacy, work policies, and physician status. This was consistent with the findings of a recent questionnaire-based study that assessed graduate pharmacists’ perspectives on an undergraduate ethics course [35]. Confidentiality, beneficence, and patients’ consent were reported to be the guiding ethical principles in professional practice.

### Theme 5: moral obligation

The participants had different opinions regarding moral obligations. The sense of duty and responsibility to respect their teachers was more dominant in the responses of 4^th^ year students as compared to the 5^th^ year students. In contrast, the sense of duty and responsibility to follow the pharmacist code of ethics, as well as religious belief and obligation, were more pronounced in the responses of 5^th^ year students. This may be because senior students and recent graduates are more likely to see respect for the profession as a moral obligation. Hattingh’s study that aimed to explore the ethical reasoning of practicing pharmacists and pharmacy interns found that the interns working in the same environment felt more obliged to respect the patients and tried to answer their questions [14]. They also admited they were engulfed by the feeling of guilt because of their action might put future patients at risk of harm due to incompetency.

### Theme 6: signposting

Another theme that was reflected in participants’ responses in our study was that of student signposting, although we found very few studies to support this theme. Our students seemed to be very much aware of not only the importance of whistleblowing but also which resources to refer to for help and incident reporting in the university as well as in the clinical practice. This finding, however, is inconsistent with that of Hattingh et al. who reported in their exploratory study that the interns were less likely to consult professional help when faced with an ethical dilemma and more likely to act on their own accord, as compared to the community pharmacists [15].

### Theme 7: moral engagement and patient safety

From the participants’ responses in our study, it can be deduced that they are very eager to help and engage with others in need. The participants stated that by engaging more with patients, they can influence their health in a better way. This may be a result of early exposure to the healthcare profession and compulsory summer clinical placements in early years in our PharmD program as we aim to inculcate patient safety in our students from very early years in the university. According to a white paper developed by the American Pharmaceutical Association Academy of Students of Pharmacy and the American Association of Colleges of Pharmacy Council of Deans, early exposure to pharmacists working during their practice has an impact on the students’ ethical reasoning and engagement with the profession. This led to the recommendation to educators to encourage student involvement in professional organizations [36]. However, such an early exposure without a fundamental understanding of moral engagement may do the students more harm when they face a real ethical dilemma [37].

### Implications of our study in light of existing literature

Our study has highlighted many ethical issues, as evident in the description and discussion of the themes above, which can be iterated in the pharmacy curriculum for the moral development of undergraduate students. The concept of incorporating ethical education into healthcare degree programs has emerged during the last few decades [38]. Many educational organizations have outlined the significance of ethical training, and several interventional strategies have been developed and recognized to influence students’ ethical reasoning and support their professional transition. These strategies, derived from the literature, will be discussed in this section.

The environment of the current education system encourages and promotes “convergent thinking.” This implies that obtaining the agreed-upon correct answer is more important than debating and questioning these answers [39]. This aligns with Kohlberg’s cognitive moral development theory that represents the conventional thinking level. This may explain the lack of mature ethical reasoning in the students. As the profession advances, the need to develop students’ ethical reasoning is more critical than ever [33]. Furthermore, the favorable impact of educational courses encompassing ethical reasoning has been reported in several studies [35, 38–40]. Pharmacy programs should, therefore, revise the content of their courses to develop students’ ethical reasoning, along with clinical reasoning, in the interest of producing more mature graduates with sound reasoning.

While the concept of conventional ethical courses and activities is not new, allowing the students to participate in these activities actively is. This is achieved by increasing student engagement through peer discussions, problem-solving, and role-playing. The most important trait of these activities is that it requires feedback on the teachers' part allowing teachers also to play a more active role in the process. Learners must not only complete tasks but also discuss with the peers and the teachers and construct their reasoning behind the questions being stated. This was iterated by Latif in one of his studies, which concluded that discussions between peers can foster moral development [41]. The use of the active learning methods to develop ethical reasoning in pharmacy students is, therefore, paramount, and the results will reflect in their professional practice.

A few tools to measure students' moral compass have been developed. One of these is the Situational Judgement Test (SJT), which assesses students' judgmental standards [42]. This test measures students' non-academic attributes and skills. It presents them with dilemmas that they may encounter in the workplace and requires them to judge the appropriateness of the action and its consequences. This can be incorporated into the pharmacy curriculum since it provides students with a safe environment to develop their decision-making skills using ethical reasoning.

Using social media to improve ethical reasoning is another novel idea that has been demonstrated to engage students in discussions and debates [40]. Compared to traditional teaching methods such as face-to-face lectures, it creates a space for wider discussions with peers and allows thoughtful conversations to take place between participants from diverse professions and with varied experiences [43].

### Strengths and limitations of the study

This topic has hardly been addressed and studied scientifically. Our study successfully developed ten model scenarios representing ethical conflicts. The scenarios covered different facets and range from university education to professional practice in order to have a holistic and cultural picture of the students’ professional and ethical judgement when a series of scenarios are presented to them, which they may face or might have faced in their journey of being a student through to a professional pharmacist.

The scenarios used in this study were aimed to explore students’ ethical reasoning from being student through to real-life practice even though the ethical aspects during university education might not be comparable to those in direct patient contact in professional practice. The scenarios also covered the general ethical aspects which the students may face in the university that could be applicable to students from other healthcare disciplines. The ethical aspects during a university study also appealing to other biomedical and allied health programs too.

Further research is necessary to demonstrate how this ethical reasoning can be translated to situational judgment in real life. Courses encompassing moral engagement and ethical reasoning that engage students in more and better active learning techniques can be of benefit in developing their situational judgment. Objective Structured Clinical Examinations (OSCE) and SJT can help assess students' ethical reasoning and situational judgment. There is also a further need for more research to quantitively assess or measure how students deal with moral or ethical dilemmas and the impact on learning outcomes and professional practice.

## Conclusion

Healthcare professional, in particular a pharmacist’s ability to rationalize and judge is contingent on the prior experience, exposure and education. The evaluation of ethical aspects in the current and future professional practice of undergraduate pharmacy students has revealed that social and professional responsibility, academic integrity, legal obligation, moral obligation, and moral engagement and patient safety are the ethical reasoning affecting their situational judgment. Student engagement is also affected by the state of mind and feelings of mutual trust, perceived cultural influence and peer pressure. The students were prone to seek help from university administrators or teachers when faced with situations in which they were helpless.

## Supplementary Information

**Additional file 1.** Ethical dilemmas developed and validated in the study.

## Declarations

### Availability of data and materials

The datasets used and analyzed in this study can be available from the corresponding author on reasonable request.
