# Supplementary material for: Situational judgment using ethical reasoning in Saudi undergraduate pharmacy students
Source: BMC Med Ethics. 2022 Apr 12;23:42. doi: 10.1186/s12910-022-00768-x (PMC9006411; doi:10.1186/s12910-022-00768-x)
Supplement: Supplementary file 1 — Additional file 1. Ethical dilemmas developed and validated in the study. [file 12910_2022_768_MOESM1_ESM.docx]

**Situational Judgment using Ethical Reasoning in Undergraduate Pharmacy Students**

**Supplementary information 1**

Scenarios Developed by the Team to Capture Students’ Situational Judgment

| **Scenarios** |
| --- |
| 1. You are a 3^rd^ year pharmacy student and there are 38 students in your class. Your best friend in the class cannot afford to be absent from any class as he/she is already short of attendance and any more absence will affect his/her eligibility to sit for the exam. He/she sends you a message on WhatsApp this morning before the class that he/she slept late at night and cannot ‘come out of bed due to tiredness’ to come to the university. He/she requests you to sign his/her attendance on the attendance sheet during the class. How would you deal with this situation? [UNDERGRADUATE] |
|  |
| 2. You are a 4^th^ year pharmacy student currently taking your online/virtual training. You receive the link for one of the daily online quizzes in the morning. You work hard to find out the answers for the quiz and submit them. Your best friend calls you and says that he woke up very late and did not have enough time to find out the answers to the quiz. He/she asks you for all the answers. What will you do in this situation? [UNDERGRADUATE] |
|  |
| 3. You find out that one of your classmates has made an insulting comment on Twitter about one of the doctors of the university. How will you react in this situation? [UNDERGRADUATE] |
|  |
| 4. You are at your on-site pharmacy training. You have some doubts about some of the professional practice of your supervisor/preceptor. On several occasions, you see her taking medication that is returned from the wards or by patients for her personal use. You are very concerned about this unprofessional practice, but you are very hesitant about discussing this with her because she still has to sign your evaluation form. How would you deal with this situation? [UNDERGRADUATE] |
|  |
| 5. You are a 5^th^ year student now. You have an assignment for a course where you are required to work on a given case study as a group. As a group leader, you divide the task among group members. Everybody has done their research and send their work to you. You are now putting together the assignment by combining everybody’s work. You notice that one of your colleagues has just copied all his/her given tasks from the Wikipedia website and has not put any effort into it at all. If you add this work to the assignment, the tutor will deduct the mark of the whole group due to plagiarism. What would you do in this situation? [UNDERGRADUATE] |
|  |
| 6. You are a newly qualified pharmacist now working in a hospital. The mother of your very close friend from school gets admitted to the hospital due to abdominal pain, weakness, and blood in the stool. The doctor diagnoses her with colorectal cancer. However, she does not want to let her family know at this stage that she has cancer as they would be ‘worried’. Your friend is persisting and asking about what is wrong with his/her mother. What would you tell your friend? [POSTGRADUATE] |
|  |
| 7. You have switched your job and now working in a community pharmacy (or OPD in hospital). A patient comes to you with a new prescription for an antiepileptic. He has been prescribed this for the first time and wants to know about the side effects he might experience. However, you believe that telling him about the possible side effects might put him off taking the medicine altogether, which could significantly affect his health. What would you do in this situation? [POSTGRADUATE] |
|  |
| 8. In the same job position, you are approached by a patient without any prescription and he has extreme pain in the lower back at the moment. He asks for tramadol capsules. You explain to him that this medicine cannot be given without a prescription. The patient tells you that he has a prescription, but he has lost it and he cannot wait to book an appointment with the doctor to have the new prescription. How would you deal with this situation? [POSTGRADUATE] |
|  |
| 9. You have just started your residency (or other relevant training) in the United States. Your first rotation is in Obstetrics and Gynecology Ward. You realize that some of the patients are admitted for termination of pregnancy in the first or second trimester. As a Muslim, you do not feel comfortable in dispensing or checking medication that would be used for such terminations because of religious belief. What would you do in this situation? [POSTGRADUATE] |
|  |
| 10. You are back in Saudi Arabia now after achieving training/qualification abroad. You have just started a ‘smoking cessation clinic’ in your community pharmacy (or OPD in hospital) in which the smokers are advised how to quit smoking and what products are available to help them stop smoking. You notice that one of your colleagues who is also selected to provide this service to patients was just smoking outside this morning from where the patients enter the pharmacy waiting area. You have a serious concern that this does not leave a good impression on patients and believe that pharmacists must ‘lead by an example’. What would you do in this situation? [POSTGRADUATE] |

**Supplementary information 2**

Quotes from Participants’ Responses Supporting the Themes

| **Theme 1: Student engagement** |
| --- |
| “[They] might be having problems at home or [were] having anxiety or depression. I truly believe that the student’s state of mind matter, it might affect his attendance and marks but that doesn’t make [them] any less worthy of education or a less ambitious student. I wish if the university pay more attention to evaluate the students.” |
| **Theme 2: Social and professional responsibility** |
| “… the patient has the right to maintain privacy information.” |
| “I will tell him he has the right to know what wrong with his mother.” |
| “It is my duty to inform the patient.” |
| “I will help my colleague to quit smoking because he also needs help.” |
| **Theme 3: Academic integrity** |
| “I will help [them] explain what [they] does not understand, because it will benefit [them].” |
| “I will help to find the right answer but I will not give [them] all answer. Except if there no time to answer these questions.” |
| “Talk to [them] and tell that what [they] did was wrong.” |
| “would advise [them] to redo it without any plagiarism or copy.” |
| **Theme 4: Legal obligation** |
| “Tell [them] No because it is not ethical thing and it is illegal.” |
| **Theme 5: Moral obligation** |
| “The doctor of the university He has the right to respect.” |
| “What happened is immoral and he should be good example for all trainees, including me.” |
| “[I] will tell my friend that what [they] did is not ethical and against our morals.” |
| “I won’t tell her because this is patient privacy.” |
| **Theme 6: Signposting** |
| “Discuss with head office [about what to do].”  “He doesn't have references I will go to the doctor and explain everything.”  “If I don't I [will] tell the head of the pharmacy seeking for advice.”  “Ask another pharmacist to tell her about her mother's condition.”  “Talk to my supervisors at the university and tell him the subject And I will hear his advice.”  “I will tell the preceptor of our college and ask him what I should do.” |
| **Theme 7: Moral engagement and patient safety** |
| “… but then I will state again that this drug is essential for his health and that we wouldn’t risk his health for anything.”  “… give an alternative to the patient until the appointment comes and prepare the prescription.”  “I can do is advise and convince the patient and tell her in a professional and decent way that is not good.” |
